# Evidence from an Applied Health Research Question: Health Equity Profiles of Community Health Centre (CHC) and non-CHC clients

**DOI:** 10.23889/ijpds.v9i5.2905

**Published:** 2024-09-18

**Authors:** Lesley Plumptre, Eliane Kim, Angela Robertson, Jennifer Rayner, Shelly-Ann Hall, Cynthia Damba, Michael Paterson, Luke Mondor

**Affiliations:** 1 ICES; 2 Parkdale Queen West CHC; 3 Black Health Equity Working Group; 4 Alliance for Healthier Communities; 5 Ontario Health

## Objective/Approach

The Black Health Equity Working Group's Applied Health Research Question aimed to compare cancer screening rates and surgical wait times between community health centre (CHC) clients based on race-related data and non-CHC clients.

CHC client data was categorized by self-identified racial groups, with Black self-identification compared to non-Black racialized, White, and missing racial self-identification, and non-CHC clients. Health card numbers were encrypted to link individuals to the Primary Care Population dataset for breast, cervical, and colorectal cancer screening rates. Surgical wait time indicators, such as the number of patients undergoing surgery and average wait times for initial consultation and completed surgeries, were derived from the Wait Time Information System. Assessments were conducted semi-annually from fiscal year 2018 to 2021.

## Results

Following the onset of COVID-19, CHC clients self-identifying as Black experienced the most significant decrease (6.8%) in colorectal screenings compared to other groups. Mammogram screenings remained consistently higher for CHC clients self-identifying as Black pre- and post-pandemic. Average cervical cancer screening rates were approximately 8% higher among CHC clients compared to non-CHC clients, irrespective of racial self-identification. However, due to small sample sizes and missing data among self-identified racial groups, trends in surgical wait times for both CHC and non-CHC clients were unstable.

## Conclusion/Implications

Tailored interventions targeting Black CHC clients can enhance cancer screening rates, particularly colorectal screenings. The analysis of CHC data offers valuable insights into race-based disparities in health outcomes. Improved data completeness is essential for accurately assessing health outcome variations among different racial groups.

